# A division of labor in perception-action integration via hierarchical alpha-beta to beta-gamma coupling and local catecholaminergic control

**DOI:** 10.1038/s42003-026-09564-4

**Published:** 2026-01-21

**Authors:** Marida Zhupa, Christian Beste

**Affiliations:** 1 Cognitive Neurophysiology, Department of Child and Adolescent Psychiatry, Faculty of Medicine, TU Dresden, Dresden, Germany; 2 German Center for Child and Adolescent Health (DZKJ), Partner Site Leipzig/Dresden, Dresden, Germany

**Keywords:** Cognitive control, Human behaviour

## Abstract

The flexible handling of perception-action representations is crucial for cognitive control such as response inhibition, which depends on the catecholaminergic system. However, how cross-frequency interactions support perception-action integration during response inhibition, and how they are modulated by catecholamines, remains unknown. In this placebo-controlled study employing methylphenidate, using electroencephalography (EEG) and a modified Go/Nogo task, we investigate phase-amplitude coupling (PAC) between theta (θ), alpha (α), beta (β), and gamma (γ) oscillations. We demonstrate that these interactions are hierarchically organized, with early α-β PAC supporting perceptual-motor representation, and subsequent β-γ coupling refining downstream processing. Transfer entropy analyses indicate a feed-forward α-β to β-γ influence, suggesting that slower oscillations gate updates in faster bands. Crucially, methylphenidate selectively enhances late β-γ coupling, supporting a functional specialization where α-β rhythms enable access and reconfiguration, while β-γ rhythms mediate local control. These findings suggest a temporally structured mechanism where the catecholaminergic system modulates flexible perception-action integration during response inhibition.

## Abstract (web summary)

Perception-action integration depends on hierarchical phase-amplitude coupling (PAC). α-β PAC shows stronger directional dependence toward β-γ PAC. Methylphenidate selectively enhances β-γ PAC, linking catecholaminergic activity to response inhibition.

## Introduction

Neural oscillatory activity serves as a central means of information processing in the brain^1–3^, contributing to the human cognitive capacity. Elementary functions, such as perception, action, and their integration, have long been examined regarding their neural oscillatory underpinnings. More recently, attempts have been made to conceptualize neural oscillatory activity in the theta (θ), alpha (α), beta (β), and gamma (γ) ranges in accordance with cognitive science concepts on how perception-action integration occurs^4^. These influential and well-supported cognitive frameworks such as the Theory of Event Coding (TEC) and the Binding and Retrieval in Action Control (BRAC), have posited that features defining the perceptual input and features defining the motor response to incoming sensory information are stored in shared mental representations, which have been termed “event files” ^5–8^. Goal-directed acting can be seen as a process of handling these event files^6^. The concept of how activity in the theta, alpha, beta, and gamma ranges enables perception-action integration^4^ is directly based on the handling of such event files. Central to this neurophysiological concept is that neural oscillatory activity exhibits a complex, coordinated interaction that facilitates the dynamic integration of perception and action^4^. According to this concept, information stored in the event file about the interrelation of perceptual and motor codes is represented through beta band activity. When action-relevant information is presented (e.g., a stimulus signaling the inhibition of a motor response), theta-band activity and gamma-band activity are involved in the retrieval of this integrated information^4^. Crucially, this implies an interplay of theta/gamma and beta band activity. Moreover, it has been suggested that theta/gamma band activity is likely modulated by alpha band activity, because alpha-band activity is conceptualized as an inhibition-timing signal: increases and decreases in alpha power (event-related synchronization/desynchronization) reflect changes in inhibition on the binding and retrieval processes indexed by theta and gamma activity^4^. While recent work has corroborated an interplay among these frequency bands^9–11^, the temporal profile of the interplay between the different oscillatory activities remains unknown. Are there specific periods during perception-action integration in which an interplay between theta (θ), alpha (α), beta (β), and gamma (γ) activity is taking place?

One mechanism underlying the interplay of different frequencies is cross-frequency coupling^12^. Especially, phase-amplitude coupling (PAC) seems relevant because it effectively integrates activity across different spatial and temporal scales^12^, which is essential for common coding^13,14^. PAC likely enables the multiplexing of information^15^, which means that information can be represented and transmitted simultaneously by modulating different high-frequency signals at various phases of a low-frequency wave. In combination with the concept outlined above^4^, we hypothesize that variations in the handling of event files will affect (1) theta-beta coupling, (2) alpha-gamma coupling, and (3) beta-gamma coupling.

However, multiple cross-frequency couplings may be at play during the dynamic handling of integrated perception-action representations^4^. Beta band activity, for example, can be simultaneously involved in cross-frequency couplings with the theta band and the gamma band. This raises the question of whether one of the possible couplings plays a particularly prominent role, as information is not only exchanged between the theta and beta bands but also between the beta and gamma bands. PAC only provides information if there’s statistical coupling between two frequency bands, but not whether it reflects driving or being driven. It is conceivable that a PAC between two frequencies is under the control of a PAC in other frequency bands. This can be examined using transfer entropy measures, particularly the net information flow. When analyzing two time-resolved PACs, the net information flow determines whether the strength/dynamics of PAC in A carry predictive information about the strength/dynamics of PAC in B^16–18^. Since beta band activity is likely connected to multiple other frequencies during perception-action integration^4^, especially PACs involving beta band activity, are hypothesized to reveal an information flow between PACs during dynamic perception-action integration.

Considering the neurobiological basis of how PAC may affect perception-action integration, it is relevant to note that previous findings have revealed that pharmacologically modulating catecholaminergic activity affects event file processing^19–22^. Suppose PAC and the net information flow between coupled frequencies reflect a central element for the dynamic handling of perception-action representations. Therefore, we hypothesize that a pharmacological modulation of the catecholaminergic system modulates PAC and the net information flow. This is possible since evidence reveals that PAC is affected by changes in dopaminergic system activity ^23–25^.

To examine the above hypotheses, we re-used EEG data^19^ from a response inhibition task (i.e., an adapted Go/Nogo task) derived from the TEC framework. In this task, stimulus features were either shared between Go and Nogo trials (overlapping condition) or fully distinct (non-overlapping condition). Only in the overlapping condition was a reconfiguration of perceptual-motor bindings necessary to correctly inhibit responses. This paradigm has been used in previous studies targeting flexible action control^26,27^. We re-analyze the data where previous analyses have focused on time-domain activity patterns^19^.

## Results

### Behavioral results

**Fig. 1:**
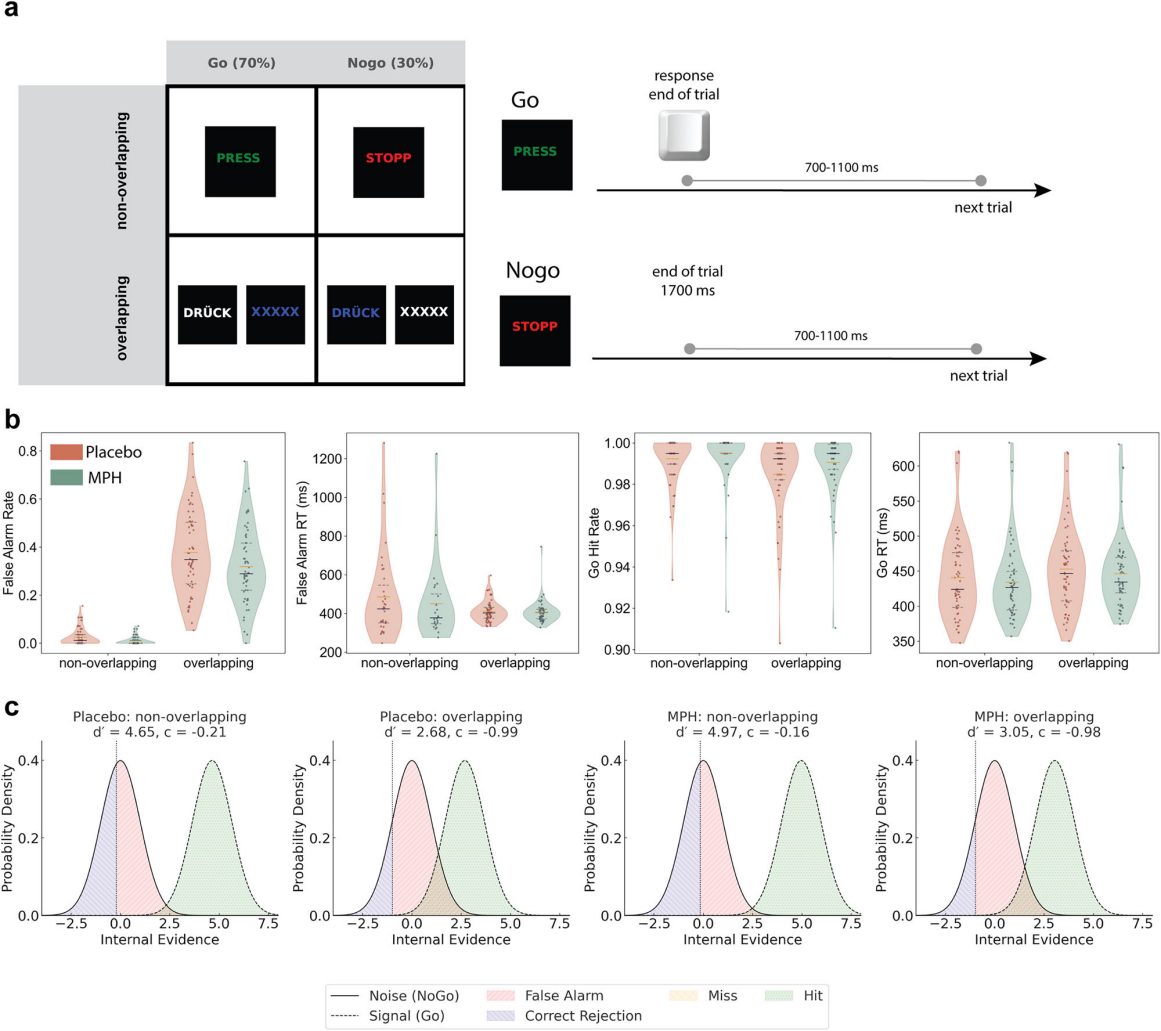
Behavioral performance in the TEC Go/Nogo task. **a** It illustrates the stimulus conditions and trial structure of the task. A 2 × 2 grid shows the specific stimuli for the non-overlapping and overlapping conditions for both Go (70% frequency) and Nogo (30% frequency) trials. The corresponding trial timelines depict a Go trial, which ends upon response, and a Nogo trial, which ends after a 1700 ms timeout. Each trial was followed by a randomly jittered inter-trial interval (ITI) of 700–1100 ms. **b** Behavioral performance metrics under Placebo (orange) and MPH (green) for each condition. From left to right: false alarm rate on Nogo trials, reaction time (RT) for false alarms, hit rate on Go trials, and Go-trial RTs. Overlapping trials showed higher false alarm rates and slower Go RTs than non-overlapping trials. MPH reduced false alarm rates in both conditions, with minimal effects on RTs. Within each violin plot, the mean is indicated by a gold line, and the median by a deep blue line. **c** Signal detection modeling of performance. Distributions show internal evidence strength for signal (Go) and noise (Nogo) trials, fitted separately for each condition and drug session. Overlapping trials (right) showed reduced sensitivity (d′) and more liberal response bias (c) compared to non-overlapping trials (left). MPH improved sensitivity (higher d′) in both conditions, with no significant effect on decision bias.

The TEC Go/Nogo task manipulated inhibitory control demands by varying the degree of stimulus overlap: non-overlapping cues were easy to discriminate, whereas overlapping cues shared features across Go and Nogo stimuli and increased interference (see Fig. 1a; for a complete description of the task, refer to the Methods section). Analyses primarily focused on condition-related differences in task performance, with drug effects evaluated using paired comparisons since all participants completed both sessions. Under Placebo, false alarm rates were substantially lower in the non-overlapping condition (*M* = 0.025, SD = 0.035) than in the overlapping condition (*M* = 0.377, SD = 0.173; *t*(57) = 17.66, *p* < 0.001); the same pattern emerged under MPH, with errors increasing from the non-overlapping (*M* = 0.010, SD = 0.017) to the overlapping condition (*M* = 0.319, SD = 0.157; *t*(57) = 15.42, *p* < 0.001) (Fig. 1b). Direct drug comparisons (MPH vs. placebo) showed that MPH significantly reduced false alarm rates in the overlapping condition (*t*(57) = −5.17, *p* < 0.001), and to a lesser extent also in the non-overlapping condition (Wilcoxon signed-rank: *z* = −3.62, *p* < 0.001, *r* = −0.48). Reaction times (RTs) of false alarms also varied: under Placebo, false responses were slower in the non-overlapping (*M* = 486 ms, SD = 229) than in the overlapping condition (*M* = 408 ms, SD = 61; Wilcoxon signed-rank: *z* = −2.14, *p* = 0.033, *r* = −0.36), whereas under MPH this RT difference was not significant (non-overlapping: *M* = 451 ms, SD = 203; overlapping: *M* = 407 ms, SD = 81; *p* = 0.422). False alarm RTs did not differ significantly between drug conditions in either trial type (non-overlapping: *p* = 0.818; overlapping: *p* = 0.725) (Fig. 1b).

Go-trial performance also showed clear effects of stimulus overlap. Under Placebo, hit rates were slightly but significantly reduced in the overlapping compared with the non-overlapping condition (*M* = 0.992, SD = 0.012 vs. *M* = 0.985, SD = 0.018; Wilcoxon signed-rank: *z* = −5.62, *p* < 0.001, *r* = −0.74), and the same pattern emerged under MPH (*M* = 0.995, SD = 0.013 vs. *M* = 0.991, SD = 0.015; *z* = −5.66, *p* < 0.001, *r* = −0.74). Go-trial RTs showed the opposite pattern, with faster responses in the non-overlapping than in the overlapping condition under both Placebo (*t*(57) = −4.54, *p* < 0.001) and MPH (*t*(57) = −5.13, *p* < 0.001), and no significant drug differences in either condition (all *p* > 0.3) (Fig. 1b).

To further characterize task performance, we computed sensitivity (d′) and response bias (c) from corrected hit and false-alarm rates, as previously done in the Go/Nogo task^28^. Signal detection analyses confirmed that stimulus overlap impaired discrimination performance. d′ was significantly reduced in overlapping compared to non-overlapping trials under both Placebo (*M* = 2.68, SD = 0.65 vs. *M* = 4.65, SD = 0.57) and MPH (*M* = 3.05, SD = 0.71 vs. *M* = 4.97, SD = 0.47), indicating impaired perceptual separation of Go and Nogo stimuli. MPH increased d′ in both conditions, reflecting enhanced discrimination under the drug (see Fig. 1c).

Response bias (c) was consistently liberal (negative) across conditions and became more liberal in the overlapping trials (Placebo: *M* = −0.21 → −0.99; MPH: *M* = −0.16 → −0.98). However, no reliable drug effect on c was observed in either trial type (non-overlapping: *p* = 0.314; overlapping: *p* = 0.886), likely because MPH simultaneously increased hit rates and reduced false alarms, balancing out overall decision bias (see Fig. 1c).

In sum, overlapping stimuli markedly increased error rates, but MPH improved inhibitory control by reducing false alarms, particularly under the more demanding overlapping condition. These effects replicate prior findings in the same task^19^.

### Phase-amplitude coupling

We first examined whether the amplitude of higher-frequency activity was systematically modulated by the phase of slower rhythms, quantified using Tort’s modulation index^29^. Analyses focused on Nogo trials in overlapping and non-overlapping conditions, separately for Placebo and MPH sessions.

**Fig. 2:**
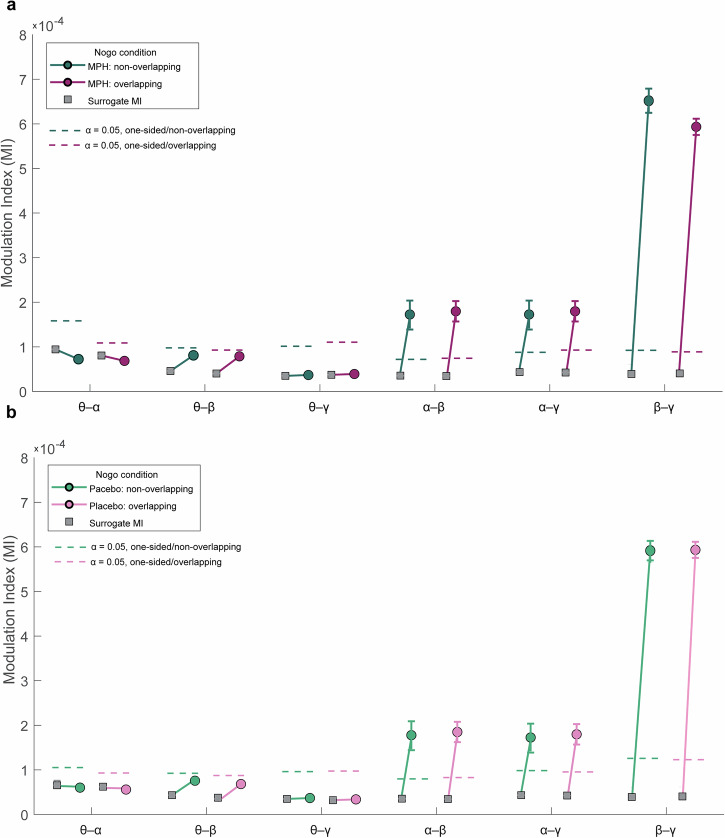
Real and surrogate modulation indices (MI) across band pairs in Nogo conditions. **a** MPH session; **b** Placebo session. Modulation indices (MI) are shown for phase-amplitude coupling across the frequency band pairs (θ-α, θ-β, θ-γ, α-β, α-γ, β-γ) under two Nogo conditions: non-overlap (teal) and overlap (pink). For each pair and condition, the mean surrogate MI (phase-shuffled null in gray square) and real MI (circle) are plotted. Error bars indicate ± s.e.m. across participants (*n* = 58). The dashed line marks represent the one-sided *α* = 0.05 surrogate threshold used to assess whether observed MI exceeds chance, color-coded to match the condition.

As shown in Fig. 2a, b, the strongest cross-frequency coupling was observed for β-γ, followed by weaker but reliable α-β and α-γ interactions, whereas θ-band couplings were negligible (θ-band couplings did not survive FDR correction, *q* ≥ 0.05). Specifically, β-γ modulation indices (MIs) were highest under both Placebo (≈5.8–5.9 × 10⁻⁴) and MPH (≈6–7 × 10⁻⁴), consistently exceeding the 95% surrogate threshold across trial types, and survived FDR correction for multiple comparisons (*p* < 0.001 for all β-γ conditions). α-β coupling, although smaller in magnitude (Placebo ≈1.1–1.2 × 10⁻⁴; MPH ≈ 1.5–1.7 × 10⁻⁴, *p* < 0.001), also reliably exceeded surrogate confidence limits, indicating statistically reliable coupling and surviving FDR correction (*p* < 0.001). The same was observed for α-γ interactions, which were similarly low in amplitude (Placebo ≈1.0–1.2 × 10^−^⁴; MPH ≈ 1.1–1.3 × 10⁻⁴, *p* < 0.001), yet remained consistently above the 95% surrogate threshold and survived FDR correction (*p* < 0.001). In contrast, θ-band couplings, including θ-β (≈5–8 × 10⁻⁵), remained weak and did not exceed surrogate thresholds in any condition (all *p* > 0.25). Together, these results demonstrate that cross-frequency interactions during response inhibition are primarily driven by α-, β-, and γ-band couplings, with no detectable contribution from θ-band activity.

### Time-resolved phase-amplitude coupling

To capture the temporal dynamics of cross-frequency interactions, Modulation Index (MI) was recomputed in sliding 100 ms windows advanced every 40 ms, yielding time courses for the frequency pairs that showed significant effects in the initial analysis. Condition differences were tested with cluster-based permutation procedures^30^ (see “Statistics and reproducibility”).

**Fig. 3:**
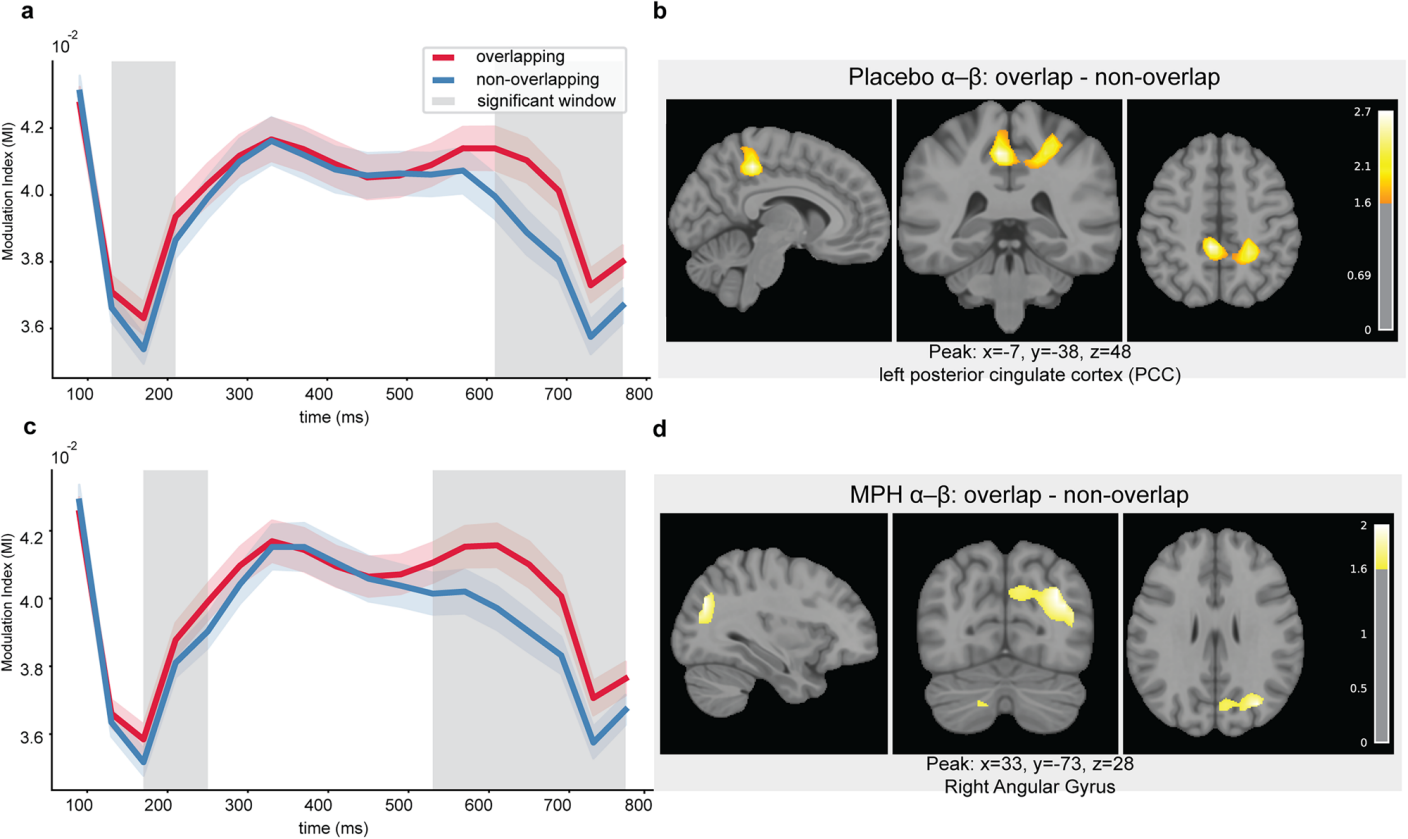
Time-resolved α-β MI and corresponding source localization. Temporal profiles of α-β MI on Nogo trials under Placebo (**a**) and MPH (**c**). Red and blue lines show group-mean MI for overlapping and non-overlapping conditions, respectively; shaded ribbons indicate ± s.e.m. across participants (*n* = 58). Gray vertical bands mark time clusters with significant condition differences identified by cluster-based permutation testing. Source localization of the overlap-non-overlap contrast for α-β MI within the significant time windows under Placebo (**b**) and MPH (**d**). Maps show sagittal, coronal, and axial slices of volumetric *t*-statistics derived from minimum-norm estimates, thresholded for display as in Methods. Color bars indicate *t*-values; text annotations show peak MNI coordinates and anatomical labels.

Within the Placebo session, α-β MI differed between overlapping and non-overlapping trials in two windows: 130–210 ms (*p* = 0.011) and 610–770 ms (*p* = 0.001) (Fig. 3a). Under MPH, α-β MI again showed biphasic differences at 170–250 ms (*p* = 0.019) and 530–770 ms (*p* = 0.001) (Fig. 3c). Regarding α-γ there was no significant drug effect or difference between overlapping and non-overlapping conditions (see Supplementary Fig. 2).

**Fig. 4:**
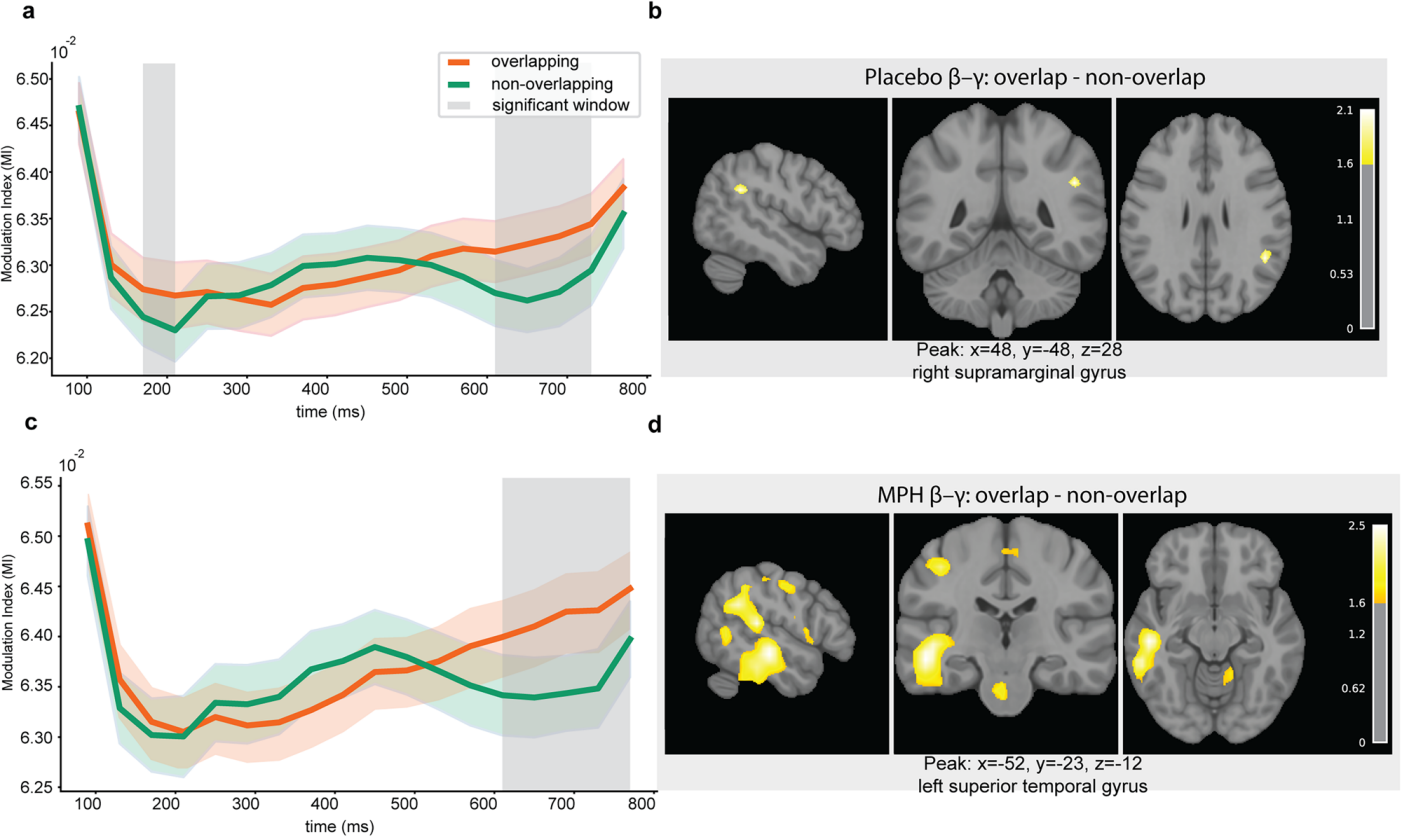
Time-resolved β-γ MI and corresponding source localization. Temporal profiles of β-γ MI on Nogo trials under Placebo (**a**) and MPH (**c**). Red and blue lines show group-mean MI for overlapping and non-overlapping conditions, respectively; shaded ribbons indicate ± s.e.m. across participants (*n* = 58). Gray vertical bands mark time clusters with significant condition differences identified by cluster-based permutation testing. Source localization of the overlap–non-overlap contrast for β-γ MI within the significant time windows under Placebo (**b**) and MPH (**d**). Maps display sagittal, coronal, and axial slices of volumetric *t*-statistics derived from minimum-norm estimates, thresholded for display as described in Methods. Color bars indicate *t*-values; captions show peak MNI coordinates and anatomical labels.

For β-γ MI, Placebo showed differences at 170–210 ms (*p* = 0.043) and 610–730 ms (*p* = 0.004) (Fig. 4a), whereas under MPH a difference emerged only in the later window, 610–770 ms (*p* = 0.001) (Fig. 4c).

**Fig. 5:**
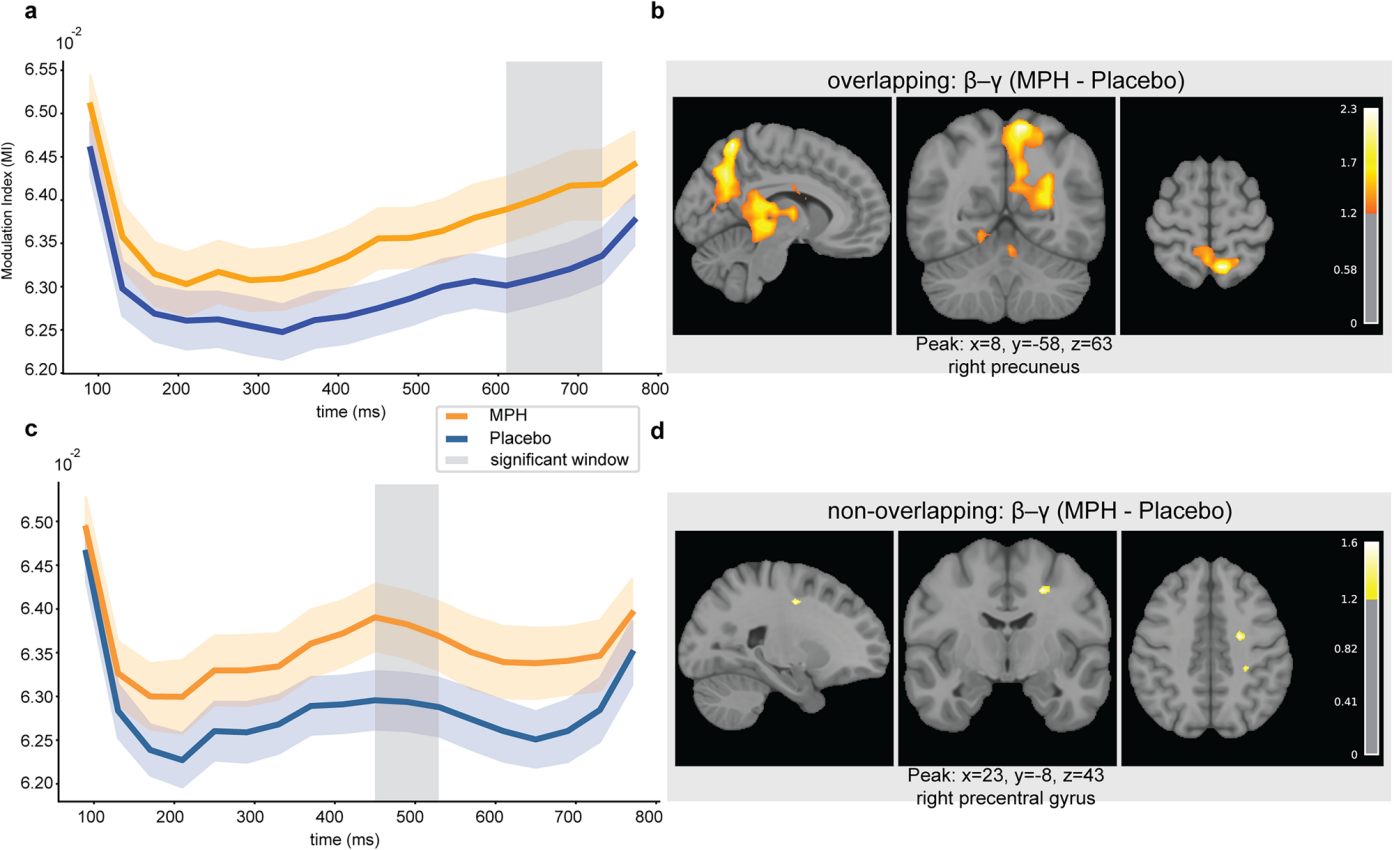
Between-drug differences in β-γ MI and corresponding source localization. Time courses of β-γ MI on Nogo trials, contrasting MPH (orange) and Placebo (blue), for overlapping (**a**) and non-overlapping (**c**) conditions. Lines show group-mean MI; shaded ribbons indicate ± s.e.m. across participants (*n* = 58). Gray vertical bands mark time clusters with significant MPH-Placebo differences identified by cluster-based permutation testing. Source localization of the MPH-Placebo contrast for β-γ MI within the significant time windows for overlapping (**b**) and non-overlapping (**d**) trials. Maps show sagittal, coronal, and axial slices of volumetric *t*-statistics from minimum-norm estimates, thresholded for display as described in Methods. Color bars indicate *t*-values; captions report peak MNI coordinates and anatomical labels.

Direct drug comparisons revealed that in overlapping trials, β-γ MI was stronger under MPH than Placebo at 610–730 ms (*p* = 0.037) (Fig. 5a), while α-β showed no significant drug effect. In non-overlapping trials, β-γ MI differed between drugs at 450–530 ms (*p* = 0.042) (Fig. 5c), again with no significant α-β effect, as shown in Supplementary Fig. 1.

In summary, α-β MI consistently exhibited a biphasic temporal profile (early ~130–250 ms, late ~530–770 ms), whereas β-γ MI effects were predominantly late ( ~ 600–770 ms). MPH selectively modulated β-γ but not α-β coupling. A direct comparison of α-β and β-γ coupling strength, based on time-averaged MI values from significant windows, is reported in the Supplementary Information (see Supplementary Fig. 4). These analyses confirmed significantly stronger β-γ coupling across all drug and task conditions (all *p* < 0.001), with overlapping trials generally yielding higher PAC than non-overlapping trials.

To directly examine whether MPH-induced changes in β-γ coupling were related to behavioral improvements, we correlated the difference in PAC (MPH—placebo) with the corresponding difference in false alarm rates. For overlapping trials, β-γ MI was extracted from the late window (610–730 ms) that showed a significant drug effect. Individual PAC differences did not correlate with behavioral differences (*r* = 0.07, *p* = 0.603), and regression analysis confirmed the absence of a linear association (*β* = 6.08, *p* = 0.603). For non-overlapping trials, β-γ MI differences were taken from the earlier window (450–530 ms), which also showed a drug effect. Again, no reliable relationship emerged between neural and behavioral changes (*r* = −0.05, *p* = 0.740; *β* = −0.66, *p* = 0.740). Together, these analyses indicate that although MPH consistently enhanced β-γ coupling, the magnitude of this neural modulation varied independently from behavioral improvements across individuals.

### Source localization

To identify the cortical localization of significant PAC effects, sensor-level results were projected into source space using minimum-norm estimates^31^ constrained to the cluster-significant time windows (Figs. 3–5). This yielded volumetric maps of modulation index differences for the α-β and β-γ pairs that showed condition effects.

For α-β MI, the placebo contrast (overlap–non-overlap) mapped to posterior midline cortex, including PCC/precuneus, and bilateral superior parietal regions, with additional lateral prefrontal contributions (Fig. 3b). Under MPH, sources were more right-lateralized and centered on angular gyrus/precuneus, with a cerebellar contribution (Fig. 3d). For β-γ PAC, placebo effects localized mainly to right temporoparietal cortex (including inferior parietal/supramarginal gyrus) and posterior midline (precuneus) (Fig. 4b). Under MPH, a broader bilateral network was engaged with left temporo-parietal dominance (inferior parietal, including angular/supramarginal), extending into sensorimotor areas, thalamus, and right frontal/precuneus regions (Fig. 4d). The between-drug contrast for β-γ revealed posterior medial parietal involvement (bilateral precuneus) in overlapping trials, alongside thalamic/striatal, sensorimotor, and occipito-temporal contributions; in non-overlapping trials, effects were more focal and centered on right pre/postcentral cortex (Fig. 5b, d). Across contrasts, inferior parietal/angular gyrus and posterior midline (PCC/precuneus) appeared consistently. Full coordinate lists (t-values, MNI, AAL labels^32^; local maxima separated by ≥10 mm) are provided in Supplementary Table 1.

### Transfer entropy

**Fig. 6:**
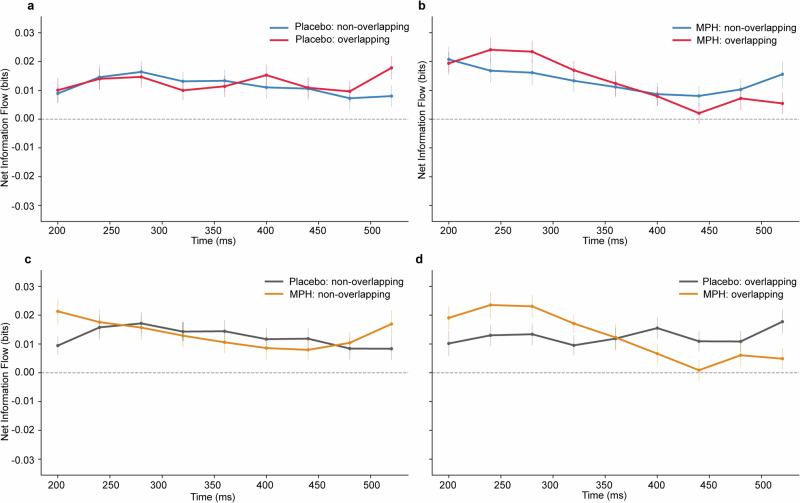
Directionality of PAC interactions quantified with transfer entropy. Net transfer entropy (NetTE) time courses within each drug session for non-overlapping (blue) and overlapping (red) trials under Placebo (**a**) and MPH (**b**). NetTE time courses comparing drugs for non-overlapping (**c**) and overlapping (**d**) trials (Placebo, gray; MPH, orange). NetTE is defined as TE(α-β → β-γ) -TE(β-γ → α-β); positive values indicate a dominant influence from α-β to β-γ. Points correspond to the centers of sliding analysis windows; error bars denote ± s.e.m. across participants (*n* = 58). The dashed horizontal line marks zero net information flow (bits).

To assess the directionality of PAC interactions as measured by the Modulation Index (MI), we computed net transfer entropy (NetTE), defined as the difference between α-β → β-γ and β-γ → α-β information flow^33–35^. This measure captures whether the past of one PAC process improves prediction of the other, with positive NetTE indicating feed-forward dominance of α-β over β-γ. Paired, cluster-based permutation tests revealed no significant differences between overlapping and non-overlapping trials within either drug session (Fig. 6a, b), and no significant differences between MPH and Placebo within either condition type (all contrasts: no clusters, two-tailed *α* = 0.05, 1000 permutations) (Fig. 6c, d). In contrast, one-sample cluster tests against zero demonstrated a robust positive NetTE, indicating dominant α-β → β-γ influence across sessions and conditions. Under Placebo, significant positive clusters were observed from 200–520 ms in both non-overlapping (cluster *p* = 0.001) and overlapping trials (cluster *p* = 0.001). Under MPH, NetTE clusters were again positive, spanning 200–520 ms in non-overlapping trials (*p* = 0.001) and 200–400 ms in Overlapping trials (*p* = 0.001). No negative clusters (β-γ → α-β dominance) were detected in any condition, see Fig. 6.

Together, these findings reveal a consistent mid-latency feed-forward dominance of α-β over β-γ PAC ( ~ 200–520 ms). This directional hierarchy was stable across task contexts (overlapping–non-overlapping) and was not detectably altered by MPH at the group level under the present analysis settings.

## Discussion

**Fig. 7:**
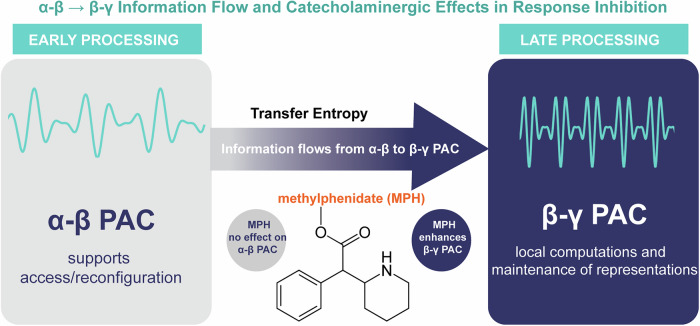
Conceptual summary of PAC dynamics and catecholaminergic modulation during response inhibition. This schematic summarizes the key findings of the current study: α-β PAC supports early-stage access or reconfiguration of perception-action representations, while β-γ PAC reflects later local computations and representational maintenance. Information flow analyses revealed directional influence from α-β to β-γ PAC, consistent with a hierarchical model of temporal coordination. Methylphenidate selectively enhanced β-γ PAC but did not affect α-β PAC or directional flow. The figure illustrates the conceptual interpretation of these findings and does not display empirical data.

The current study investigated how phase-amplitude coupling (PAC) and information flow between oscillatory frequency bands (theta, alpha, beta, and gamma) facilitate the dynamic integration of perception and action (Fig. 7). Specifically, the study aimed at (1) characterizing the temporal dynamics of cross-frequency couplings (θ-β, α-γ, α-β, β-γ), (2) determining the directionality of information exchange between these PACs using transfer entropy, and (3) assessing the influence of catecholaminergic modulation (via pharmacological manipulation) on PACs and net information flow. To achieve this, we employed a response inhibition task and re-analyzed data from a previous study by our group^19^.

The behavioral data reveal a well-known pattern in which response inhibition performance (reflected by the rate of false alarms) was compromised in the overlapping Nogo trials, compared to the non-overlapping Nogo trials. This reflects the fact that in overlapping Nogo trials, a reconfiguration of integrated perception-action codes is necessary^19^.

### Hierarchical cross-frequency coupling is central for perception-action integration

On the neurophysiological level, the data revealed that PAC was evident across all time points in α-β, α-γ, β-γ, suggesting that across-frequency coordination is evident during perception-action integration. Notably, differences between occasions requiring a reconfiguration of integrated perception-action codes (i.e., overlapping trials) and not requiring a reconfiguration of integrated perception-action codes (i.e., non-overlapping trials) were only evident for α-β and β-γ PAC. Here, PAC was stronger for overlapping than non-overlapping trials, which suggests that α-β and β-γ PAC are essential when integrated perception-action codes are reconfigured. Interestingly, these α-β and β-γ PAC modulations were evident in early time windows ( ~ 200 ms after stimulus presentation) and towards the end of the analyzed time windows (from ~600 ms onwards). Previous studies have consistently shown that power/amplitude modulations in the θ, α, and β frequency bands occur between the mentioned time windows^9,10,19,27,36^. Thus, α-β and β-γ PAC precede and succeed power modulations in the frequency bands, also giving rise to perception-action integration processes^4^. According to a recent conception^4^, θ/γ activity is likely essential for the retrieval/reconfiguration of integrated perception-action representations, the content of which is possibly handled through β-band activity^37^. The retrieval is triggered by the presentation of a stimulus^6^ (i.e., at the start of the analyzed time window), and α-band associated attentional selection or top-down controlled processes become activated. Crucially, since β-band processes likely store the content of perception-action representations^4,38–40^, it is important to have direct access to this information, especially when perception-action representations have to be reconfigured. In that case, the content to be modified may best become reconfigured if there is a direct relation between β-band processes and processes reflected by α and γ activity. Precisely this is reflected by the obtained α-β and β-γ PAC modulations at the beginning of the analyzed time window. At the end of the analyzed time window, the same direction of modulation was evident. While here, no retrieval processes can be in charge because the cognitive processes have already been completed (as evidenced by the behavioral data); the cognitive system must be reset. Re-iterant processes are at the core of the dynamical handling of perception-action representations^6^. As such, the α-β and β-γ PAC modulations from ~600 ms onwards may reflect the reset of processes initiated during the retrieval phase. Consequently, this reset reflects a similar modulatory pattern across both overlapping and non-overlapping conditions, as observed during the retrieval time interval. For all of these processes, the data show that inferior parietal, posterior cingulate and inferior parietal regions are involved. The inferior parietal/angular gyrus regions emerge because they are the neurocognitive hubs of feature binding and attentional integration^39^, where α-β interactions are essential. The posterior cingulate cortex (PCC) emerges because of its role in resetting and updating cognitive states^40^. Together, these regions provide the large-scale cortical architecture that supports both retrieval/reconfiguration (early) and reset/re-iterant processing (late) of perception-action codes^4^.

Nevertheless, α-β and β-γ PAC are constantly evident during the entire processing cascade (i.e., also between 200 and 600 ms after stimulus presentation). During that time interval, the α-β PAC was larger than the β-γ PAC in both overlapping and non-overlapping conditions, suggesting that the interplay between α-β is more critical for perception-action integration processes than the interplay between β-γ. Intriguingly, the analysis of the net information flow also suggests that α-β PAC plays a central role in perception-action integration processes. Through the net information flow analysis, we examined whether there is an asymmetry in directional dependence from the slower α-β PAC to the faster β-γ PAC, which would be consistent with a hierarchical coordination of different cross-frequency coupling mechanisms during perception-action integration. The results revealed a clear directional pattern in the net information flow with α-β PAC more strongly predicting subsequent β-γ PAC than vice versa. This hierarchical pattern is compatible with recent spatiotemporal accounts proposing that brain activity is structured across hierarchically nested temporal scales, where slower dynamics provide a temporal context for faster, more local processes, as described in the Dynamic Layer Model of the brain^41^ and, more broadly, in Common Currency Theory, which characterizes neural-mental relations in terms of temporo-spatial correspondences across multiple timescales^42^.

As discussed above, β-band dynamic plays a central role in perception-action integration because it likely stores the content of perception-action representations^4,43,44^. The finding that α-β PAC shows stronger directional dependence towards β-γ PAC reflects a general principle evident in both overlapping and non-overlapping trials. This supports the central role of β-band activity as an organizing “hub frequency” in perception-action integration processes. The β-band possibly acts as an interface between different other frequencies (i.e., α and γ) involved in perception-action integration.

### A catecholaminergic basis for PAC during perception-action integration

The last aim of the study was to examine whether PAC and hierarchical PAC are based on catecholaminergic system activity. We modulated catecholaminergic system activity through methylphenidate, a combined dopamine (DA) and norepinephrine (NE) transporter blocker^45^, which has previously been shown to modulate power-based EEG correlates of perception-action integration^19–22^. Previous analyses using the same dataset demonstrated that catecholaminergic modulation alters perception-motor integration by changing the stability of representational content during response inhibition^19^.

Often, the actions of a pharmacological modulation of the catecholaminergic system (i.e., DA + NE) have been conceptualized through gain modulation principles^46^. Gain control modulates the signal-to-noise ratio (SNR) in neural circuits^47^, and an increased gain control is associated with greater precision of neural processes. Higher gain control, as induced through the administration of methylphenidate, has recently been shown to alter directed communication in cortical networks during the anticipation of action-induced perceptual effects^21^. The current findings provide further evidence that increases in catecholaminergic system activity alter neural communication, as demonstrated by changes in cross-frequency coupling profiles. Again, mostly the late time intervals showed modulatory effects, and effects were evident for overlapping and non-overlapping trials. Interestingly, only β-γ PAC was modulated, and also the net information was unaffected by the pharmacological modulation. Thus, catecholamines play roles in the modulation of neurophysiological processes underlying perception-action integration. The reasons why β-γ PAC was modulated may be manifold: First, β-γ PAC is linked to local computations—e.g., maintaining active sensory-motor representations, gating inputs, stabilizing representational contents^38,48^. These functions are sensitive to neuromodulatory gain-control^49,50^. Catecholamines can adjust the local gain and stability of cortical assemblies, as reflected in nested β-γ activity^51^. Cross-PAC coordination may subserve more “architectural” or integrative roles. These dynamics may depend more on structural connectivity and not moment-to-moment neuromodulator tone. Second, DA/NE effects operate on slow timescales (hundreds of milliseconds to seconds)^46,52,53^, which may be ideal for modulating the gain of local β-γ circuits. Cross-PAC interactions, as delineated in the net information flow, often require precise phase-phase alignment across regions in tens of ms. These faster dynamics may be beyond the temporal resolution of catecholaminergic modulation, relying instead on fast inhibitory/excitatory circuits and conduction delays (possibly GABA or glutamate are more important in this context). Although MPH increased β-γ PAC and improved response-inhibition at the group level, these effects did not correlate across individuals. This likely reflects non-linear brain-behavior relationships such as inverted-U neuromodulatory effects^46^.

## Conclusion

In summary, we showed that phase-amplitude coupling (PAC) is a central organizing principle of perception-action integration. Crucially, only α-β and β-γ PAC distinguished conditions that required reconfiguring perception-action codes. These PAC modulations occurred both early ( ~ 200 ms, during retrieval) and late ( ~ 600 ms, during reset), suggesting that PAC supports both the initiation and reinitialization of perception-action processes. Net information flow analyses revealed a directional hierarchy in which α-β PAC drives β-γ PAC, highlighting α-β as an upstream integrative mechanism and β-γ as a local stabilizer of representational content. This establishes the β-band as a hub frequency. A modulation of the catecholaminergic system affected β-γ PAC but not the hierarchical α-β → β-γ flow. Taken together, the findings identify a division of labor: α-β → β-γ PAC implements hierarchical control, unlikely affected by catecholaminergic modulation, while β-γ PAC supports local representational computations tuned by neuromodulatory gain. The study offers an account of how flexible perception-action integration emerges from the interplay between hierarchical cross-frequency coupling and catecholaminergic control.

## Materials and methods

### Participants

The final sample comprised 58 healthy adults (31 males, 27 females; mean age = 23.72 years, SD = 2.55; range = 20–30 years). Participants were drawn from a larger cohort of volunteers taking part in an ongoing research project at TU Dresden. For the present analyses, 60 individuals were randomly selected from this cohort; two were excluded because pervasive EEG artifacts resulted in an insufficient number of artifact-free trials for reliable behavioral and EEG analyses, yielding a final sample of 58 participants. This sample size in a within-subject cross-over design is comparable to, or larger than, those of closely related methylphenidate and Go/Nogo or perception-action integration studies, which typically include approximately 30–45 healthy participants such as^20,54–56^.

All participants were screened to rule out neurological or psychiatric disorders using a brief telephone interview and standardized assessments, including the Adult Self-Report for Ages 18–59 (ASR)^57^ and the Mehrfachwahl-Wortschatz-Intelligenztest (MWT-B)^58^. The ASR was used to obtain dimensional indices of psychological symptoms and to identify indications of clinically relevant psychopathology, and the MWT-B to characterize verbal intellectual functioning. In this randomly selected subsample, ASR and MWT-B scores did not indicate clinically relevant psychopathology or intellectual impairment, and no participant was excluded on this basis.

The procedures were approved by the Ethics Committee of the Medical Faculty of TU Dresden. All ethical regulations relevant to human research participants were followed. Written informed consent was obtained from all participants prior to participation, and participants received monetary compensation. Parts of this dataset have been reported in previous publications addressing different research questions^9,11,13,27,36^.

### MPH administration

This study was conducted using a randomized, double-blind, cross-over design^59^. Each individual participated in two separate experimental sessions, with a time interval between them of at least 24 h and no more than 14 days (mean: 5.02 days, SD = 3.32). Participants were randomly assigned to one of two groups. The MPH-first group (*n* = 29) received methylphenidate (MPH) during the first session and a placebo during the second, while the placebo-first group (*n* = 29) received the placebo first and MPH second. The groups did not differ in age, gender distribution, or the time between sessions. Neither participants nor researchers knew the administration order. In the MPH session, a single dose of immediate-release MPH (0.25 mg/kg body weight) was given, in line with previous pharmacological EEG studies in healthy young adults by our group, where this moderate dose has been shown to reliably modulate conflict- and learning-related adjustments in cognitive control while remaining within a well-tolerated clinical range^19,56,60^. An indistinguishable placebo was used in the other session. Experimental tasks began about 75 min after administration to align with peak MPH plasma levels^61–63^.

### Task

Participants performed a TEC Go/Nogo task established by Chmielewski and Beste^26^ (Fig. 1a), designed to examine the effects of MPH on perception-action integration in the context of response inhibition by manipulating the degree of feature overlap between Go and Nogo stimuli. Stimuli were presented in German on a 17-inch CRT monitor positioned 60 cm in front of the participants. Prior to the main experiment, participants received written and verbal instructions and completed 30 practice trials that included both overlapping and non-overlapping conditions to ensure familiarization. Each trial began with a fixation cross presented for 700–1100 ms (jittered), followed by a stimulus shown for 450 ms. Participants had up to 1700 ms from stimulus onset to respond; if no response was given, the trial terminated.

The task comprised Go and Nogo trials, each occurring in either non-overlapping or overlapping conditions. To induce a prepotent response tendency, Go and Nogo trials were presented in a 7:3 ratio within each condition, yielding 196 Go and 84 Nogo trials per condition. The task was divided into seven blocks of equal length. Within each block, all trial types occurred equally often, and their order was pseudorandomized. Participants were instructed to respond to Go trials by pressing the space bar and to withhold responses on Nogo trials. In the non-overlapping condition, Go stimuli consisted of the word PRESS (green) and Nogo stimuli of the word STOPP (red). In the overlapping conditions, Go stimuli were either the word DRÜCK (“press”) in white or a string of five blue letters (XXXXX). The corresponding overlapping Nogo stimuli were DRÜCK in blue or XXXXX in white. These conditions created systematic feature overlap between Go and Nogo stimuli, requiring reconfiguration of previously established event files, a manipulation known to increase false alarm rates in Nogo trials.

### EEG recording and preprocessing

Electroencephalographic (EEG) data were recorded during the TEC Go/Nogo task using a 60-channel passive Ag/AgCl electrode cap (EasyCap Inc.). The ground electrode was located at coordinates *θ* = 58°, *ϕ* = 78°, and signals were referenced online to the FCz electrode. Electrode impedances were maintained below 5 kΩ throughout the recording session. Data were sampled at 500 Hz using BrainVision Recorder software (v2.1). Offline preprocessing was performed in MATLAB (R2020a) using the EEGLAB toolbox^64^. Data were downsampled to 256 Hz and band-pass filtered between 1 and 100 Hz using a zero-phase FIR filter. A 50-Hz notch filter was applied to remove line noise. Noisy or flat channels were detected and removed with the clean_rawdata() plugin; on average, 3.0 (SD = 2.2) channels per participant were excluded. Signals were then re-referenced to the common average. To attenuate high-amplitude, transient artifacts (e.g., head movements, electrode pops), Artifact Subspace Reconstruction (ASR)^65^ was applied with a standard deviation cutoff of 15. Segments that could not be reconstructed were discarded. Subsequently, an extended Infomax Independent Component Analysis (ICA) was run on the continuous data. Independent components were automatically classified using ICLabel^66^, and components labeled as ocular, muscle, or cardiac artifacts with >0.85 probability were visually confirmed and removed. Removed channels were reconstructed by spherical-spline interpolation. Epochs containing voltage fluctuations exceeding ±100 μV at any electrode were rejected from further analysis.

Finally, data were segmented into epochs from −200 to 1000 ms relative to stimulus onset. Baseline correction was applied using the −200 to 0 ms interval.

### Phase-amplitude coupling

As an initial step, we quantified cross-frequency coupling at the sensor level using Tort’s Modulation Index (MI), which measures the extent to which the amplitude of a faster oscillation is modulated by the phase of a slower one^29^. Analyses focused on Nogo trials in the overlapping and non-overlapping conditions, separately for each session (Placebo and MPH).

For each participant, EEG data were filtered into predefined phase- and amplitude-frequency bands (θ: 4–7 Hz, α: 9–12 Hz, β: 13–30 Hz, γ: 31–100 Hz) using zero-phase finite impulse response (FIR) filters in MNE-Python^67^. The analytic signals were obtained via the Hilbert transform, yielding instantaneous phase and amplitude time series. We examined all pairwise combinations of low- and high-frequency bands relevant to phase-amplitude coupling. PAC was estimated separately for each electrode channel. Within each channel, trials were concatenated along the time axis, and MI was computed once per condition and band-pair. The low-frequency phase (0–360°) was divided into 18 equal bins (20° each), and the mean high-frequency amplitude in each bin was used to construct an amplitude distribution *P*.

$${KL}\left(P,U\right)={\sum }_{j=1}^{18}{P}_{j}\cdot \log \left(\frac{{P}_{j}}{{U}_{j}}\right)$$

The MI was computed as the normalized Kullback-Leibler divergence between *P* and the uniform distribution *U*:

$${MI}=\frac{{KL}\left(P,U\right)}{\log \left(18\right)}$$

This procedure served to identify which band-pairs showed significant PAC at the sensor level. Only those band-pairs were subsequently analyzed in the time-resolved PAC and source-localization steps.

### Time-resolved PAC

Time-resolved PAC was computed with a fixed 100 ms window advanced every 40 ms (60% overlap) for α-β, α-γ and β-γ coupling, ensuring ≥1 cycle of the phase rhythm across pairs ( ≈ 0.9–1.3 cycles for α at 9–12 Hz; ≥1.3–3 cycles for β at 13–30 Hz) and maintaining comparability of temporal resolution across conditions. This window length balances temporal resolution with the requirement to capture at least one full cycle of the lowest phase frequency, which is a standard criterion for obtaining stable phase estimates and PAC measures^68^. The 40 ms step (60% overlap) was chosen as a moderate overlap within the range typically used in EEG sliding-window analyses ( ≈ 50–75% overlap)^68–71^. Results were unchanged when excluding the 9-Hz edge.

### Source localization

Source reconstruction was performed in a volumetric space using FreeSurfer’s fsaverage template with a 5-mm grid ( ≈ 13,222 voxels)^72^. The EEG forward model used a three-layer BEM, with a minimum source-sensor distance of 5 mm. Epoched data (−200 to 800 ms; baseline −200 to 0 ms) were analyzed with a depth-weighted minimum-norm estimate and dSPM output (*λ*² = 1/9; loose orientation = 1; depth weighting = 0.8). Condition-wise evoked responses were computed for overlapping and non-overlapping trials (overlapping pooled across the two overlapping Nogo cues), separately for Placebo and MPH sessions; individual volumetric source estimates were obtained in fsaverage space. Time windows for source-level PAC were predefined from the sensor-level time-resolved PAC analysis: only cluster-significant windows (family-wise error controlled across time by a cluster-based permutation test)^30^ were taken forward. Within each predefined window (concatenating multiple significant segments where applicable), voxel-wise phase-amplitude coupling was quantified using Tort’s Modulation Index^29^ as described in the “Phase-amplitude coupling” section. This yielded one volumetric MI map per subject, condition, and band pair. These source-level PAC maps were used descriptively to visualize the generators of the cluster-corrected sensor-level effects and were not subjected to an additional voxel-wise multiple-comparison procedure.

### Transfer entropy

Directed interactions between α-β and β-γ PAC were quantified using transfer entropy (TE), an information-theoretic, model-free measure of time-asymmetric dependence^33–35^. TE tests whether the past of one process improves the prediction of the present of another beyond what the target’s own past explains, thereby providing a direction-specific index of information flow without assuming linear coupling. In this study, TE assessed whether slower α-β PAC dynamics tend to drive faster β-γ PAC dynamics, and vice versa.

For each subject and condition, channel-level PAC estimates were averaged to yield one α-β and one β-γ time series per trial. Trial-wise PAC values were computed in 100 ms windows advanced every 40 ms (0–800 ms epoch), yielding 18 samples per trial for each PAC pair. TE was then calculated on sliding 400 ms windows (10 consecutive PAC samples, shifted by one sample), which balances temporal resolution with local stationarity and provides sufficient past-present transitions for stable estimation. This produced 9 TE estimates per subject at centers from 250–570 ms post-stimulus. TE was estimated in the discrete formulation with history length *k* = 1. MI values were discretized into three equiprobable bins using subject-specific, time-invariant quantile edges to ensure stable occupancy across the epoch. Within each window and trial, we estimated the joint distribution over the states *(*${y}_{t},{y}_{t-1},{x}_{t-1}$) where *X* and *Y* denote the symbolized α-β and β-γ PAC time series, respectively. The transfer entropy from *X* to *Y* was defined as the conditional mutual information between the past of *X* and the present of *Y*, given the past of *Y*:

$${{\mbox{T}}}{{\mbox{E}}}_{{\mbox{X}}\to {\mbox{Y}}}={{\mbox{I}}}\left({X}_{t-1};{Y}_{t},|,{Y}_{t-1}\right)={\sum }_{{y}_{t},{y}_{t-1},{x}_{t-1}}p\left({y}_{t},{y}_{t-1},{x}_{t-1}\right)\log \frac{p\left({y}_{t},|,{y}_{t-1},{x}_{t-1}\right)}{p\left({y}_{t},|,{y}_{t-1}\right)}$$

This quantity captures the additional predictive value of *X*’s past beyond that contained in *Y*’s own history. Within each window, TE was computed in both directions and averaged across trials, yielding$\,{\mbox{T}}{{\mbox{E}}}_{\alpha \beta \to \beta \gamma }\,$ and ${\mbox{T}}{{\mbox{E}}}_{\beta \gamma \to \alpha \beta }$ and averaged per-trial values to obtain a subject-level time series. A net information flow index was defined as:

$${\mbox{NetTE}}\left(t\right)={\mbox{T}}{{\mbox{E}}}_{\alpha \beta \to \beta \gamma }\left(t\right)-{\mbox{T}}{{\mbox{E}}}_{\beta \gamma \to \alpha \beta }\left(t\right)$$

such that NetTE(*t*) > 0 indicates dominant α-β → β-γ influence at time *t* and NetTE(*t*) < 0 indicates dominant β-γ → α-β influence. Windows with constant symbolic sequences (no state changes) were conservatively assigned TE = 0 to avoid undefined conditional probabilities.

### Statistics and reproducibility

All analyses were performed within subjects, as each participant completed both placebo and MPH sessions. Unless otherwise noted, tests were two-tailed with *α* = 0.05. Behavioral performance (false alarm rates and reaction times of false alarms) was compared using paired *t*-tests when data met normality assumptions, and Wilcoxon signed-rank tests otherwise. Effect sizes for Wilcoxon tests are reported as rank-biserial correlations (*r*)^73^.

For PAC, statistical significance was assessed by generating surrogate null distributions through random circular shifts of the phase time series relative to the amplitude envelope^29^ (1000 surrogates per participant, condition, channel, and band-pair). One-sided *p* values were calculated as the proportion of surrogate values greater than or equal to the observed modulation index, using the correction (*k* + 1)/(*N* + 1)^74^. Within each participant, false discovery rate (FDR) correction (*q* = 0.05)^75^ was applied across channels for each condition and band-pair. Time-resolved PAC was tested using non-parametric cluster-based permutation tests^30^ (two-tailed, 1000 permutations). Adjacent samples exceeding the cluster-forming threshold of *α* = 0.05 were grouped, and cluster significance was assessed against the permutation distribution of maximum cluster statistics, thereby controlling the family-wise error rate across time.

For source localization, group statistics were computed with voxel-wise paired t-tests contrasting overlapping–non-overlapping trials within each drug session. For β-γ PAC only, additional voxel-wise t-tests contrasted MPH vs. Placebo within each condition type. No voxel-level multiple-comparison correction was applied; source reconstructions are shown to illustrate the spatial distribution of effects statistically validated at the sensor level.

Transfer entropy (TE) analyses used non-parametric cluster-based permutation tests^30^ (1000 permutations, two-tailed, *α* = 0.05) on subject-level NetTE time courses (α-β → β-γ minus β-γ → α-β). Paired tests contrasted overlapping–non-overlapping trials and MPH vs. Placebo, while one-sample tests against zero assessed directional dominance. Family-wise error across time was controlled using the cluster-based permutation procedure.

### Reporting summary

Further information on research design is available in the Nature Portfolio Reporting Summary linked to this article.

## Supplementary information

Supplemental Information

Description of Additional Supplementary File

Supplementary Data 1

reporting summary

## Data Availability

Supplementary Data 1 and the additional datasets analyzed in this study are available in the OSF repository^76^, 10.17605/OSF.IO/5UENV.

## Code availability

Further custom code used to process the data can be found here^76^: 10.17605/OSF.IO/5UENV.

## References

[1] Engel, A. K. & Singer, W. Temporal binding and the neural correlates of sensory awareness. *Trends Cogn. Sci.* **5**, 16–25 (2001). 11164732 10.1016/s1364-6613(00)01568-0

[2] Fries, P. Rhythms for cognition: communication through coherence. *Neuron* **88**, 220–235 (2015). 26447583 10.1016/j.neuron.2015.09.034 PMC4605134

[3] Varela, F., Lachaux, J.-P., Rodriguez, E. & Martinerie, J. The brainweb: phase synchronization and large-scale integration. *Nat. Rev. Neurosci.* **2**, 229–239 (2001). 11283746 10.1038/35067550

[4] Beste, C., Münchau, A. & Frings, C. Towards a systematization of brain oscillatory activity in actions. *Commun. Biol.* **6**, 137 (2023). 36732548 10.1038/s42003-023-04531-9 PMC9894929

[5] Frings, C. et al. Consensus definitions of perception-action-integration in action control. *Commun. Psychol.* **2**, 7 (2024). 39242844 10.1038/s44271-023-00050-9 PMC11332160

[6] Frings, C. et al. Binding and retrieval in action control (BRAC). *Trends Cogn. Sci.* **24**, 375–387 (2020). 32298623 10.1016/j.tics.2020.02.004

[7] Hommel, B. Event files: feature binding in and across perception and action. *Trends Cogn. Sci.* **8**, 494–500 (2004). 15491903 10.1016/j.tics.2004.08.007

[8] Hommel, B., Müsseler, J., Aschersleben, G. & Prinz, W. The Theory of Event Coding (TEC): a framework for perception and action planning. *Behav. Brain Sci.* **24**, 849–878 (2001). 12239891 10.1017/s0140525x01000103

[9] Prochnow, A., Eggert, E., Münchau, A., Mückschel, M. & Beste, C. Alpha and theta bands dynamics serve distinct functions during perception–action integration in response inhibition. *J. Cogn. Neurosci.* **34**, 1053–1069 (2022). 35258591 10.1162/jocn_a_01844

[10] Prochnow, A., Wendiggensen, P., Eggert, E., Münchau, A. & Beste, C. Pre-trial fronto-occipital electrophysiological connectivity affects perception–action integration in response inhibition. *Cortex* **152**, 122–135 (2022). 35569325 10.1016/j.cortex.2022.04.008

[11] Wendiggensen, P. et al. Interplay between alpha and theta band activity enables management of perception-action representations for goal-directed behavior. *Commun. Biol.* **6**, 494 (2023). 37149690 10.1038/s42003-023-04878-z PMC10164171

[12] Canolty, R. T. & Knight, R. T. The functional role of cross-frequency coupling. *Trends Cogn. Sci.* **14**, 506–515 (2010). 20932795 10.1016/j.tics.2010.09.001 PMC3359652

[13] Gholamipourbarogh, N. et al. Perception-action integration during inhibitory control is reflected in a concomitant multi-region processing of specific codes in the neurophysiological signal. *Psychophysiology*10.1111/psyp.14178 (2022). 10.1111/psyp.14178 36083256

[14] Gholamipourbarogh, N. et al. Evidence for independent representational contents in inhibitory control subprocesses associated with frontoparietal cortices. *Hum. Brain Mapp.*10.1002/hbm.26135 (2022). 10.1002/hbm.26135 PMC9875938 36314869

[15] Veltz, R. & Sejnowski, T. J. Periodic forcing of inhibition-stabilized networks: nonlinear resonances and phase-amplitude coupling. *Neural Comput.* **27**, 2477–2509 (2015). 26496044 10.1162/NECO_a_00786 PMC4763930

[16] Galindo-Leon, E. E., Nolte, G., Pieper, F., Engler, G. & Engel, A. K. Causal interactions between amplitude correlation and phase coupling in cortical networks. *Sci. Rep.* **15**, 11975 (2025). 40199943 10.1038/s41598-025-95306-1 PMC11978747

[17] Martínez-Cancino, R. et al. What can local transfer entropy tell us about phase-amplitude coupling in electrophysiological signals? *Entropy* **22**, 1262 (2020). 33287030 10.3390/e22111262 PMC7712258

[18] Shi, W., Yeh, C.-H. & Hong, Y. Cross-frequency transfer entropy characterize coupling of interacting nonlinear oscillators in complex systems. *IEEE Trans. Biomed. Eng.* **66**, 521–529 (2019). 29993517 10.1109/TBME.2018.2849823

[19] Eggert, E. et al. Cognitive science theory-driven pharmacology elucidates the neurobiological basis of perception-motor integration. *Commun. Biol.* **5**, 919 (2022). 36068298 10.1038/s42003-022-03864-1 PMC9448745

[20] Eggert, E. et al. Perception-action integration is modulated by the catecholaminergic system depending on learning experience. *Int. J. Neuropsychopharmacol.* **24**, 592–600 (2021). 33730752 10.1093/ijnp/pyab012 PMC8299823

[21] Mayer, J. et al. Pharmacological modulation of directed network communication and neural hubs in action–effect integration. *Int. J. Neuropsychopharmacol.* **28**, pyaf031 (2025). 40423242 10.1093/ijnp/pyaf031 PMC12143126

[22] Prochnow, A. et al. The ability to voluntarily regulate theta band activity affects how pharmacological manipulation of the catecholaminergic system impacts cognitive control. *Int. J. Neuropsychopharmacol.* **27**, pyae003 (2024). 38181228 10.1093/ijnp/pyae003 PMC10810285

[23] Andino-Pavlovsky, V. et al. Dopamine modulates delta-gamma phase-amplitude coupling in the prefrontal cortex of behaving rats. *Front. Neural Circuits* **11**, 29 (2017). 28536507 10.3389/fncir.2017.00029 PMC5422429

[24] Devergnas, A., Caiola, M., Pittard, D. & Wichmann, T. Cortical phase-amplitude coupling in a progressive model of parkinsonism in nonhuman primates. *Cereb. Cortex* **29**, 167–177 (2019). 29190329 10.1093/cercor/bhx314 PMC6294400

[25] Wang, Z., Cao, Q., Bai, W., Zheng, X. & Liu, T. Decreased phase–amplitude coupling between the mPFC and BLA during exploratory behaviour in chronic unpredictable mild stress-induced depression model of rats. *Front. Behav. Neurosci.* **15**, 799556 (2021). 34975430 10.3389/fnbeh.2021.799556 PMC8716490

[26] Chmielewski, W. X. & Beste, C. Stimulus-response recoding during inhibitory control is associated with superior frontal and parahippocampal processes. *NeuroImage* **196**, 227–236 (2019). 30991125 10.1016/j.neuroimage.2019.04.035

[27] Prochnow, A. et al. Neural dynamics of stimulus-response representations during inhibitory control. *J. Neurophysiol.* **126**, 680–692 (2021). 34232752 10.1152/jn.00163.2021

[28] Wolman, A., Lechner, S., Angeletti, L. L., Goheen, J. & Northoff, G. From the brain’s encoding of input dynamics to its behavior: neural dynamics shape bias in decision making. *Commun. Biol.* **7**, 1538 (2024). 39562707 10.1038/s42003-024-07235-w PMC11576847

[29] Tort, A. B. L., Komorowski, R., Eichenbaum, H. & Kopell, N. Measuring phase-amplitude coupling between neuronal oscillations of different frequencies. *J. Neurophysiol.* **104**, 1195–1210 (2010). 20463205 10.1152/jn.00106.2010 PMC2941206

[30] Maris, E. & Oostenveld, R. Nonparametric statistical testing of EEG- and MEG-data. *J. Neurosci. Methods* **164**, 177–190 (2007). 17517438 10.1016/j.jneumeth.2007.03.024

[31] Hämäläinen, M. S. & Ilmoniemi, R. J. Interpreting magnetic fields of the brain: minimum norm estimates. *Med. Biol. Eng. Comput.* **32**, 35–42 (1994). 8182960 10.1007/BF02512476

[32] Tzourio-Mazoyer, N. et al. Automated anatomical labeling of activations in SPM using a macroscopic anatomical parcellation of the MNI MRI single-subject brain. *NeuroImage* **15**, 273–289 (2002). 11771995 10.1006/nimg.2001.0978

[33] Schreiber, T. Measuring information transfer. *Phys. Rev. Lett.* **85**, 461–464 (2000). 10991308 10.1103/PhysRevLett.85.461

[34] Vicente, R., Wibral, M., Lindner, M. & Pipa, G. Transfer entropy—a model-free measure of effective connectivity for the neurosciences. *J. Comput. Neurosci.* **30**, 45–67 (2011). 20706781 10.1007/s10827-010-0262-3 PMC3040354

[35] Wibral, M., Vicente, R. & Lizier, J.T. *Directed Information Measures in Neuroscience* (Springer, 2014).

[36] Talebi, N. et al. Neural mechanisms of adaptive behavior: dissociating local cortical modulations and interregional communication patterns. *iScience* **27**, 110995 (2024). 39635122 10.1016/j.isci.2024.110995 PMC11615187

[37] Engel, A. K. & Fries, P. Beta-band oscillations–signalling the status quo? *Curr. Opin. Neurobiol.* **20**, 156–165 (2010). 20359884 10.1016/j.conb.2010.02.015

[38] Spitzer, B. & Haegens, S. Beyond the status quo: a role for beta oscillations in endogenous content (re)activation. *eneuro* **4**, ENEURO.0170–17.2017 (2017). 28785729 10.1523/ENEURO.0170-17.2017 PMC5539431

[39] Seghier, M. L. The angular gyrus: multiple functions and multiple subdivisions. *Neuroscientist* **19**, 43–61 (2013). 22547530 10.1177/1073858412440596 PMC4107834

[40] Leech, R. & Sharp, D. J. The role of the posterior cingulate cortex in cognition and disease. *Brain* **137**, 12–32 (2014). 23869106 10.1093/brain/awt162 PMC3891440

[41] Northoff, G. & Buccellato, A. From slow spontaneous oscillations to consciousness—dynamic layer model of brain (DLB). Comment on “Dark brain energy: toward an integrative model of spontaneous slow oscillations” by Zhu-Qing Gong and Xi-Nian Zuo. *Phys. Life Rev.* **54**, 169–172 (2025). 40714738 10.1016/j.plrev.2025.07.013

[42] Northoff, G., Buccellato, A. & Zilio, F. Connecting brain and mind through temporo-spatial dynamics: towards a theory of common currency. *Phys. Life Rev.* **52**, 29–43 (2025). 39615425 10.1016/j.plrev.2024.11.012

[43] Pastötter, B., Moeller, B. & Frings, C. Watching the brain as it (un)binds: beta synchronization relates to distractor–response binding. *J. Cogn. Neurosci.* **33**, 1581–1594 (2021). 34496371 10.1162/jocn_a_01730

[44] Pastötter, B. et al. Increased beta synchronization underlies perception-action hyperbinding in functional movement disorders. *Brain Commun.* **6**, fcae301 (2024). 39386091 10.1093/braincomms/fcae301 PMC11462440

[45] Faraone, S. V. The pharmacology of amphetamine and methylphenidate: relevance to the neurobiology of attention-deficit/hyperactivity disorder and other psychiatric comorbidities. *Neurosci. Biobehav. Rev.* **87**, 255–270 (2018). 29428394 10.1016/j.neubiorev.2018.02.001 PMC8063758

[46] Aston-Jones, G. & Cohen, J. D. An integrative theory of locus coeruleus-norepinephrine function: adaptive gain and optimal performance. *Annu. Rev. Neurosci.* **28**, 403–450 (2005). 16022602 10.1146/annurev.neuro.28.061604.135709

[47] Hauser, T. U., Fiore, V. G., Moutoussis, M. & Dolan, R. J. Computational psychiatry of ADHD: neural gain impairments across marrian levels of analysis. *Trends Neurosci.* **39**, 63–73 (2016). 26787097 10.1016/j.tins.2015.12.009 PMC4746317

[48] Barone, J. & Rossiter, H. E. Understanding the role of sensorimotor beta oscillations. *Front. Syst. Neurosci.* **15**, 655886 (2021). 34135739 10.3389/fnsys.2021.655886 PMC8200463

[49] Orekhova, E. V. et al. Neural gain control measured through cortical gamma oscillations is associated with sensory sensitivity. *Hum. Brain Mapp.* **40**, 1583–1593 (2019). 30549144 10.1002/hbm.24469 PMC6865508

[50] Weiss, E., Kann, M. & Wang, Q. Neuromodulation of neural oscillations in health and disease. *Biology* **12**, 371 (2023). 36979063 10.3390/biology12030371 PMC10045166

[51] Iskhakova, L. et al. Modulation of dopamine tone induces frequency shifts in cortico-basal ganglia beta oscillations. *Nat. Commun.* **12**, 7026 (2021). 34857767 10.1038/s41467-021-27375-5 PMC8640051

[52] Mather, M., Clewett, D., Sakaki, M. & Harley, C. W. Norepinephrine ignites local hotspots of neuronal excitation: how arousal amplifies selectivity in perception and memory. *Behav. Brain Sci.* **39**, e200 (2016). 26126507 10.1017/S0140525X15000667 PMC5830137

[53] Seamans, J. K. & Yang, C. R. The principal features and mechanisms of dopamine modulation in the prefrontal cortex. *Prog. Neurobiol.* **74**, 1–58 (2004). 15381316 10.1016/j.pneurobio.2004.05.006

[54] Groom, M. J. et al. Effects of motivation and medication on electrophysiological markers of response inhibition in children with attention-deficit/hyperactivity disorder. *Biol. Psychiatry* **67**, 624–631 (2010). 19914599 10.1016/j.biopsych.2009.09.029 PMC2845810

[55] Groom, M. J. et al. Event-related potentials in adolescents with schizophrenia and their siblings: a comparison with attention-deficit/hyperactivity disorder. *Biol. Psychiatry* **63**, 784–792 (2008). 17977520 10.1016/j.biopsych.2007.09.018

[56] Mückschel, M., Eggert, E., Prochnow, A. & Beste, C. Learning experience reverses catecholaminergic effects on adaptive behavior. *Int. J. Neuropsychopharmacol.* **23**, 12–19 (2020). 31701133 10.1093/ijnp/pyz058 PMC7064049

[57] Achenbach, T. M. Achenbach system of empirically based assessment (ASEBA). in *The Encyclopedia of Clinical Psychology* (eds Cautin, R. L. & Lilienfeld, S. O) 1–8 (Wiley, 2015).

[58] Lehrl, S. *Manual zum MWT-B: Mehrfachwahl-Wortschatz-Intelligenztest* (Spitta GmbH, 2018).

[59] Senn, S. *Cross-over Trials in Clinical Research* (Wiley, 2002).

[60] Bensmann, W., Zink, N., Roessner, V., Stock, A.-K. & Beste, C. Catecholaminergic effects on inhibitory control depend on the interplay of prior task experience and working memory demands. *J. Psychopharmacol.* **33**, 678–687 (2019). 30816793 10.1177/0269881119827815

[61] Kimko, H. C., Cross, J. T. & Abernethy, D. R. Pharmacokinetics and clinical effectiveness of methylphenidate. *Clin. Pharmacokinet.* **37**, 457–470 (1999). 10628897 10.2165/00003088-199937060-00002

[62] Challman, T. D. & Lipsky, J. J. Methylphenidate: its pharmacology and uses. *Mayo Clin. Proc.* **75**, 711–721 (2000). 10907387 10.4065/75.7.711

[63] On Behalf of the Study Group et al. A randomised, placebo-controlled, 24-week, study of low-dose extended-release methylphenidate in adults with attention-deficit/hyperactivity disorder. *Eur. Arch. Psychiatry Clin. Neurosci.* **259**, 120–129 (2009). 19165529 10.1007/s00406-008-0845-4

[64] Delorme, A. & Makeig, S. EEGLAB: an open source toolbox for analysis of single-trial EEG dynamics including independent component analysis. *J. Neurosci. Methods* **134**, 9–21 (2004). 15102499 10.1016/j.jneumeth.2003.10.009

[65] Mullen, T. et al. Real-time modeling and 3D visualization of source dynamics and connectivity using wearable EEG. in *2013 35th Annual International Conference of the IEEE Engineering in Medicine and Biology Society (EMBC**)* 2184–2187 (IEEE, 2013). 10.1109/EMBC.2013.6609968 PMC4119601 24110155

[66] Pion-Tonachini, L., Kreutz-Delgado, K. & Makeig, S. The ICLabel dataset of electroencephalographic (EEG) independent component (IC) features. *Data Brief.* **25**, 104101 (2019). 31294058 10.1016/j.dib.2019.104101 PMC6595408

[67] Gramfort, A. et al. MNE software for processing MEG and EEG data. *NeuroImage* **86**, 446–460 (2014). 24161808 10.1016/j.neuroimage.2013.10.027 PMC3930851

[68] Samiee, S. & Baillet, S. Time-resolved phase-amplitude coupling in neural oscillations. *NeuroImage* **159**, 270–279 (2017). 28757194 10.1016/j.neuroimage.2017.07.051

[69] Oran, S. & Yıldırım, E. Effects of sampling length and overlap ratio on EEG mental arithmetic task performance: a comparative study. *Gazi Univ. J. Sci.* **37**, 1718–1733 (2024).

[70] Wilson, L. E., Da Silva Castanheira, J. & Baillet, S. Time-resolved parameterization of aperiodic and periodic brain activity. *eLife* **11**, e77348 (2022). 36094163 10.7554/eLife.77348 PMC9467511

[71] Munia, T. T. K. & Aviyente, S. Time-frequency based phase-amplitude coupling measure for neuronal oscillations. *Sci. Rep.* **9**, 12441 (2019). 31455811 10.1038/s41598-019-48870-2 PMC6711999

[72] Fischl, B. FreeSurfer. *NeuroImage* **62**, 774–781 (2012). 22248573 10.1016/j.neuroimage.2012.01.021 PMC3685476

[73] Kerby, D. S. The simple difference formula: an approach to teaching nonparametric correlation. *Compr. Psychol.* **3**, 11.IT.3.1 (2014).

[74] Phipson, B. & Smyth, G. K. Permutation P-values should never be zero: calculating exact p-values when permutations are randomly drawn. *Stat. Appl. Genet. Mol. Biol.* **9**, Article39 (2010). 10.2202/1544-6115.1585 21044043

[75] Benjamini, Y. & Hochberg, Y. Controlling the false discovery rate: a practical and powerful approach to multiple testing. *J. R. Stat. Soc. Ser. B Stat. Methodol.* **57**, 289–300 (1995).

[76] Zhupa, M. & Beste, C. A division of labor in perception-action integration via hierarchical alpha-beta to beta-gamma coupling and local catecholaminergic control. OSF 10.17605/OSF.IO/5UENV (2026). 10.1038/s42003-026-09564-4 PMC12920914 41565771

