# Supplementary material for: A division of labor in perception-action integration via hierarchical alpha-beta to beta-gamma coupling and local catecholaminergic control
Source: Commun Biol. 2026 Jan 21;9:284. doi: 10.1038/s42003-026-09564-4 (PMC12920914; doi:10.1038/s42003-026-09564-4)
Supplement: Supplementary file 1 — Supplemental Information [file 42003_2026_9564_MOESM1_ESM.pdf]

Supplementary Information

A Division of Labor in Perception-Action Integration via Hierarchical alpha-beta to beta-gamma Coupling and Local Catecholaminergic Control

Marida Zhupa, Christian Beste

Supplementary Table 1 | Local maxima from volumetric source localization of PAC effect

Peaks are listed for each contrast/section with AAL region labels and MNI coordinates. Values are *t*-statistics from dSPM source estimates, reported for the cluster-significant time windows defined in the time-resolved PAC analysis.

Peak	t	X	Y	Z	Anatomical_Region (AAL Atlas)
**Placebo α-β: overlap - non-overlap**					
1	2,74	-7	-38	48	left posterior cingulate cortex (PCC)
2	2,64	18	-43	43	RightSuperior Parietal Gyrus
3	2	18	-43	73	Right Superior Parietal Gyrus (near the vertex)
4	1,96	-37	2	58	Left Middle Frontal Gyrus
5	1,96	43	22	18	Right Inferior Frontal Gyrus (IFG)
**MPH α-β: overlap - non-overlap**					
1	2	33	-73	28	Right Angular Gyrus
2	1,78	-17	-68	-47	Left Cerebellum, Lobule VI
3	1,72	8	-68	33	Right Precuneus
**Placebo β-γ: overlap - non-overlap**					
1	2,12	48	-48	28	Right supramarginal gyrus
2	1,94	8	-73	43	Right Precuneus
**MPH β-γ: overlap - non-overlap**					
1	2,49	-52	-23	-12	Left Superior Temporal Gyrus
2	2,46	-52	-33	13	Left Middle Temporal Gyrus
3	2,35	-52	-48	28	Left Supramarginal Gyrus
4	2,26	13	-43	38	Right Precuneus
5	2,23	-47	-8	43	Left Precentral Gyrus
6	2,19	-42	-23	48	Left Postcentral Gyrus
7	2,18	-12	-38	-42	Left Cerebellum, Lobule IV-V
8	2,13	8	-53	-2	Right Fusiform Gyrus
9	2,12	-37	-3	58	Left Middle Frontal Gyrus

10	2,12	18	-8	73	Right Superior Frontal Gyrus
11	2,01	-57	-63	8	Left Angular Gyrus
12	1,92	3	-33	3	Right Thalamus
**overlapping: β-γ (MPH - Placebo)**					
1	2,33	8	-58	63	Right Precuneus
2	2,21	-12	-43	53	Left Precuneus
3	2,1	3	-33	-2	Right Thalamus
4	1,97	-47	-18	8	Left Insula
5	1,92	28	-63	18	Right Middle Occipital Gyrus
6	1,77	-7	-73	-17	Left Cerebellum, Crus I
7	1,74	18	2	43	Right Precentral Gyrus
8	1,73	63	-3	3	Right Rolandic Operculum
9	1,72	18	7	23	Right Putamen
10	1,69	-12	-8	-12	Left Thalamus
11	1,59	-42	-43	-12	Left Fusiform Gyrus
12	1,53	48	7	43	Right Middle Frontal Gyrus
13	1,36	38	-23	-7	Right Middle Temporal Gyrus
14	1,35	53	-18	-27	Right Inferior Temporal Gyrus
15	1,25	-12	-83	18	Left Superior Occipital Gyrus
16	1,2	-42	2	43	Left Precentral Gyrus
**non-overlapping: β-γ (MPH - Placebo)**					
1	2,12	48	-48	28	Right Precentral Gyrus
2	1,94	8	-73	43	Right Postcentral Gyrus

Supplementary figure 1

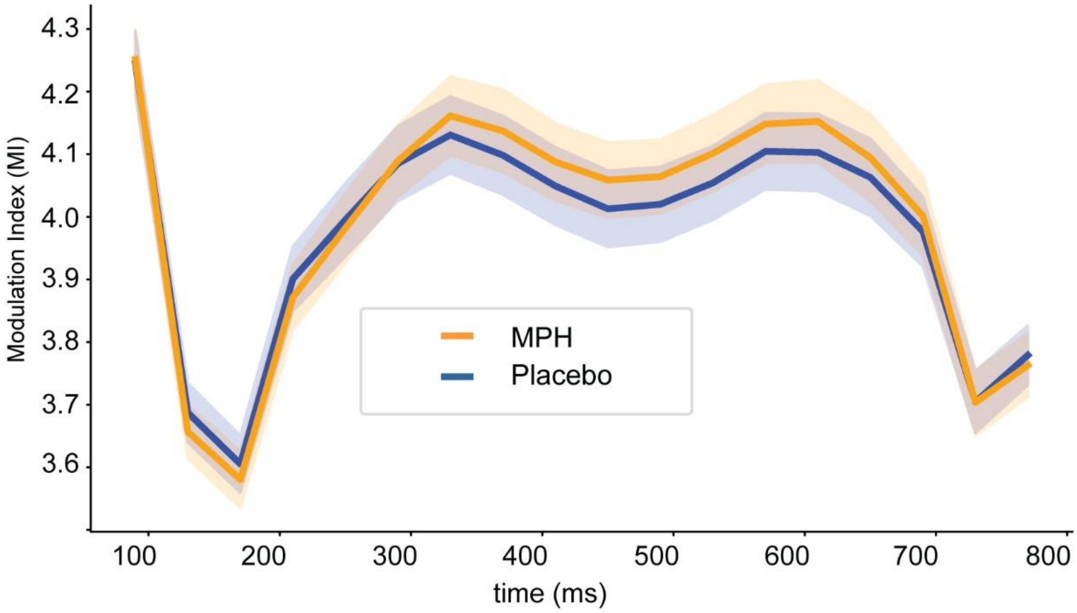

Supplementary Fig 1 | Time-resolved α-β phase-amplitude coupling (PAC) by drug session.

Group-mean Modulation Index (MI) time courses are shown for the MPH (orange) and Placebo (blue) sessions. PAC was computed with Tort's MI in sliding 100-ms windows advanced every 40 ms (60% overlap) from 100-800 ms post-stimulus. Lines indicate the mean across participants and shaded ribbons denote ± s.e.m. (n = 58). No significant drug differences were detected by two-tailed cluster-based permutation testing (1,000 permutations).

Supplementary figure 2

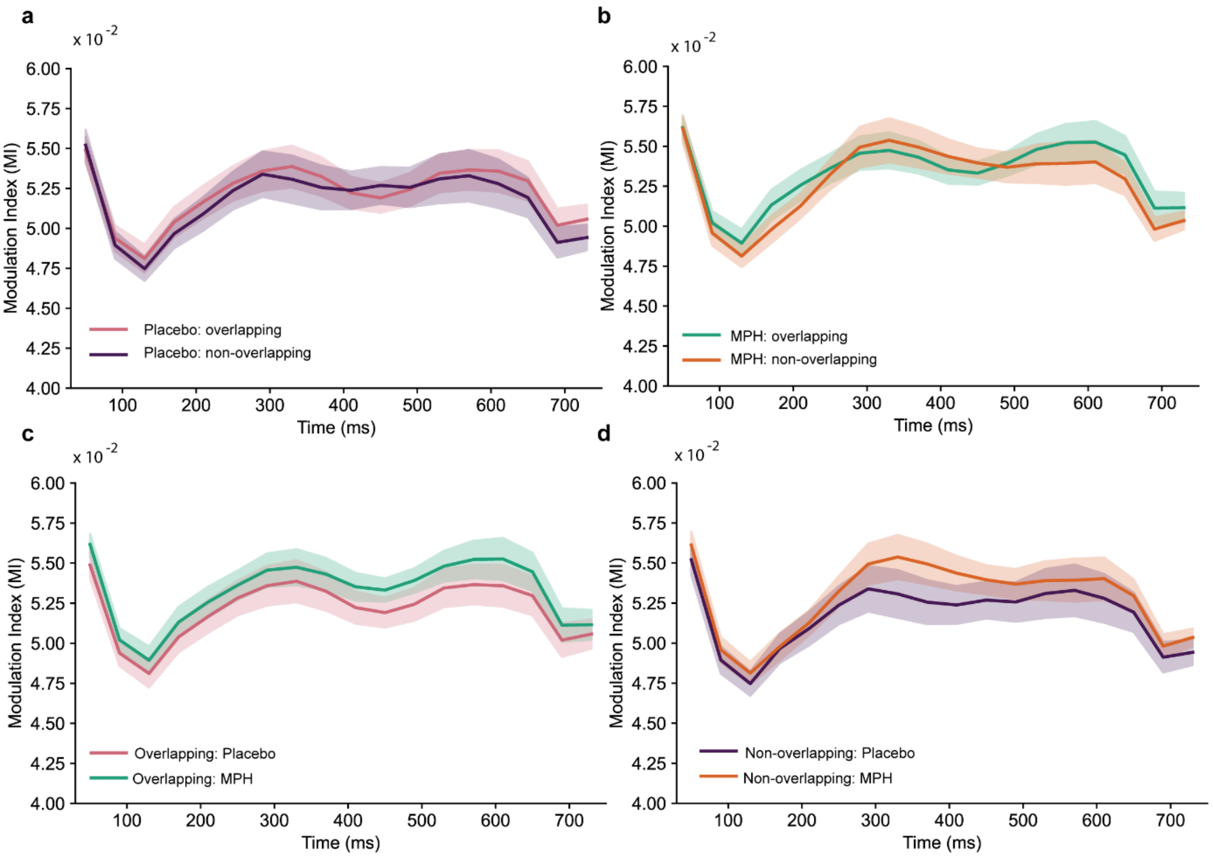

Supplementary Fig 2 | Time-resolved α-γ phase-amplitude coupling (PAC) across drug and condition.

Group-mean Modulation Index (MI) time courses for α-γ PAC are shown separately for the Placebo (panels a and c) and MPH (panels b and d) sessions. PAC was estimated using Tort's Modulation Index in 100-ms sliding windows advanced every 40 ms (60% overlap), spanning 100-800 ms post-stimulus. Lines represent participant-averaged MI values, and shaded areas indicate ± s.e.m. (n = 58). No statistically significant effects of drug or condition were detected using two-tailed cluster-based permutation testing (1,000 permutations).

Relationship Between α-β PAC and Alpha ITPC

To evaluate whether early α-β phase-amplitude coupling (PAC) could reflect stimulus-locked alpha phase alignment rather than genuine cross-frequency interactions, we quantified alpha-band inter-trial phase coherence (ITPC) in the same post-stimulus interval in which PAC effects emerged (130-250 ms). PAC values were extracted for each subject and condition (Placebo/MPH x overlap/non-overlap), and paired with the corresponding alpha ITPC estimates.

Shapiro-Wilk tests showed that all PAC variables were normally distributed (all $p \geq 0.33$), while two ITPC variables from the MPH session violated normality ($ITPC_{\text{MPH overapping}}$: $W = 0.943$, $p = 0.009$; $ITPC_{\text{MPH non-overlapping}}$: $W = 0.923$, $p = 0.001$). Because correlation analysis requires normality of both variables, associations between PAC and ITPC were assessed using Spearman rank correlations.

Across all four drug x overlap conditions, PAC-ITPC relationships were uniformly small and non-significant. Correlation coefficients ranged from $\rho = 0.10$ to $\rho = 0.24$, with corresponding p-values between 0.07 and 0.44 (Placebo: overlap: $\rho = 0.20$, $p = 0.13$; Placebo: non-overlap: $\rho = 0.21$, $p = 0.13$; MPH: overlap: $\rho = 0.24$, $p = 0.07$; MPH: non-overlap: $\rho = 0.10$, $p = 0.44$). These results indicate that variations in alpha phase alignment do not account for the condition-dependent changes in α-β PAC observed in the main analysis.

Scatterplots illustrating these relationships and the full correlations are provided in Supplementary Fig 3.

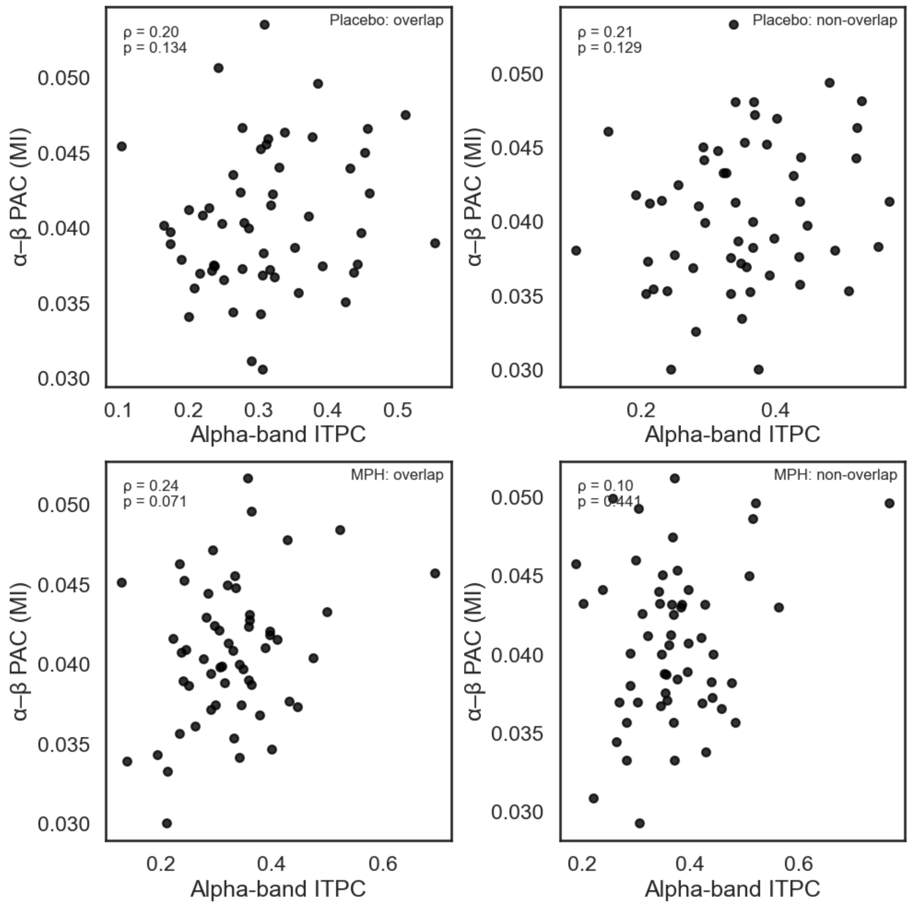

Supplementary Fig 3 | Relationship between α-β phase-amplitude coupling (PAC) and alpha-band ITPC (130-250 ms). Scatterplots show, for each condition, the association between alpha-band inter-trial phase coherence (ITPC) and α-β PAC modulation index (MI)

across participants. Conditions are separated by drug (Placebo, MPH) and feature-overlap (overlap, non-overlap). For each panel, Spearman's rank correlation coefficient (ρ) and corresponding p-value are provided. No correlation reached statistical significance in any condition, indicating that α-β PAC effects are not explained by alpha-band phase alignment at stimulus onset. Axes are scaled identically across subplots to allow direct visual comparison.

Comparison of α-β vs. β-γ coupling strength

To compare the relative strength of α-β and β-γ phase-amplitude coupling (PAC), we extracted individual modulation index (MI) values averaged across the time windows that showed significant effects in the time-resolved analyses. For each participant, MI values were computed separately for each PAC pair (α-β and β-γ), drug session (Placebo, MPH), and condition (overlapping, non-overlapping).

These values were then entered into paired-sample t-tests to directly assess whether β-γ coupling was stronger than α-β within each drug × condition combination. Across all groups, β-γ coupling was significantly stronger than α-β (Placebo: overlapping $t = -59.66$, $p < 0.001$; non-overlapping $t = -58.89$, $p < 0.001$; MPH: overlapping $t = -60.54$, $p < 0.001$; non-overlapping $t = -68.20$, $p < 0.001$), confirming the overall dominance of β-γ PAC across both drug states and task conditions.

We also tested for differences between overlapping and non-overlapping trials within each PAC pair. For α-β coupling, MI values were significantly higher for overlapping trials compared to non-overlapping ones, both under Placebo ($t = 7.00$, $p < 0.001$) and MPH ($t = 6.40$, $p < 0.001$). A similar but weaker pattern was observed for β-γ coupling, with overlapping trials yielding slightly higher MI values than non-overlapping trials under both Placebo ($t = 4.23$, $p < 0.001$) and MPH ($t = 2.47$, $p = 0.017$).

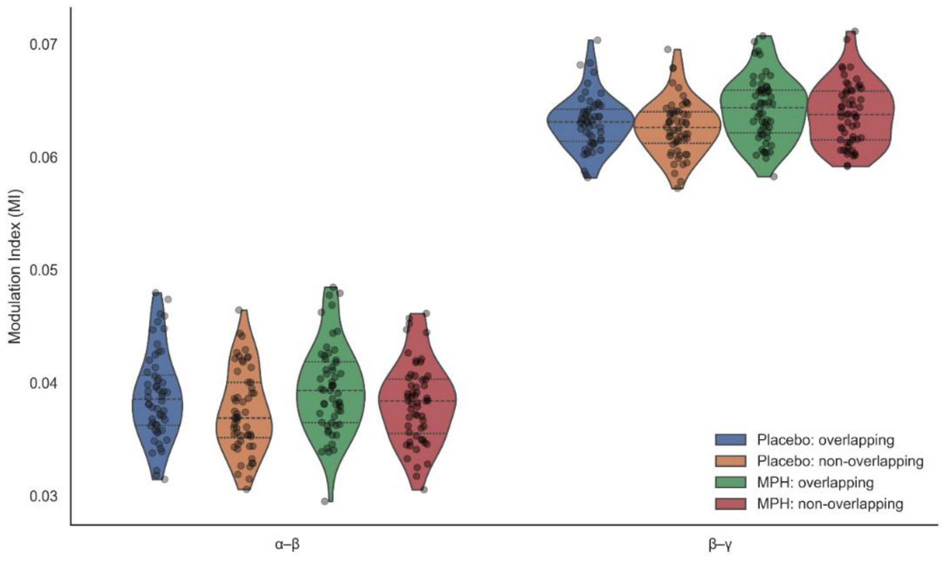

Supplementary Fig 4 | Comparison of α-β and β-γ Modulation Index (MI) across drug and condition. Violin plots show time-averaged MI values for α-β and β-γ coupling, separately for overlapping and non-overlapping trials under Placebo and MPH. Each dot represents an individual participant. MI values were averaged across significant time windows identified in the time-resolved analysis (see Methods). Across all conditions, β-γ PAC was significantly stronger than α-β PAC ($p < 0.001$, paired t-tests within each group). This pattern was consistent across drugs and task conditions.
