# Supplementary material for: A division of labor in perception-action integration via hierarchical alpha-beta to beta-gamma coupling and local catecholaminergic control
Source: Commun Biol. 2026 Jan 21;9:284. doi: 10.1038/s42003-026-09564-4 (PMC12920914; doi:10.1038/s42003-026-09564-4)
Supplement: Supplementary file 2 — Description of Additional Supplementary File [file 42003_2026_9564_MOESM2_ESM.pdf]

Description of Additional Supplementary Files

File name: Supplementary Data 1

Description: numerical source data for Figure 3, 4, 5
